# Effect of increased oxytocin doses on the mode of delivery in obese primiparous women with spontaneous or induced labour. A double-blind, randomised, controlled trial (PROXYMA)

**DOI:** 10.1186/s12884-026-08772-4

**Published:** 2026-02-11

**Authors:** Julie Carrara, Alexandre J. Vivanti, Franck Pizzagalli, Mélanie Chastrusse, Jean Bouyer, Alexandra Benachi

**Affiliations:** 1 Service de gynécologie-obstétrique, AP-HP, Hôpital Antoine Béclère, Clamart, 92140 France; 2 Université Paris Saclay, Le Kremlin-Bicêtre, 94276 France; 3 Direction de la Recherche Clinique et de l’Innovation (DRCI) de l’AP-HP, Hôpital Saint-Louis, Paris, 75010 France; 4 Université Paris-Saclay, UVSQ, Inserm, CESP, Villejuif, 94807 France

**Keywords:** High-dose oxytocin, Obesity, Caesarean section, Labour, Morbidity

## Abstract

**Background:**

The prevalence of obesity in pregnancy is increasing worldwide, and it is associated with significant morbidity during labour and delivery. A benefit of increased doses of infused oxytocin during labour in obese women has been suggested by retrospective or underpowered studies. Thus, we aimed in a randomized double-blind controlled study at evaluating whether an increased oxytocin dose (compared to a standard dose) can reduce the rate of caesarean section without increasing maternal or neonatal morbidity.

**Methods:**

PROXYMA is a French national multicentre, randomized, double-blind, controlled trial conducted in 14 maternity units. Adult primiparous obese women (body mass index ≥ 30 kg/m^2^) with singleton pregnancy, spontaneous or induced onset of labour, cephalic presentation and term, ≥ 37 weeks of gestation, will be included after informed consent. Exclusion criteria are medical contra-indication for oxytocin or hypersensitivity, previous coagulation disorders, foetal growth restriction (inferior to 5th percentile), foetal major malformation or heart rate abnormalities before use of oxytocin (at the time of inclusion), scarred uterus of gynaecological origin, planned caesarean section before labour, or severe renal failure. Patients will be randomized in a 1:1 ratio and receive either high-dose (experimental group) or standard-dose (control group) oxytocin from prescription until delivery, with increments at least every 20 min, and oxytocin will be stopped instantly at birth. The inclusion of the patient and randomization of the oxytocin dose to be administered will take place at the time of the oxytocin prescription. An “out-of-protocol” healthcare professional (midwife / nurse) will be in charge of the preparation of the treatment, in order to ensure the double-blind design. The primary outcome will be the caesarean section rate in each group. Secondary outcomes include the comparison of maternal, foetal and neonatal-related complications during labour and birth. We aim at including 882 women based on a reduction in caesarean section rate of 7% in the experimental group, with a power of 80% and an alpha risk of 5%.

**Discussion:**

Comparing two different oxytocin doses will provide clinicians with better knowledge of labour management in obese primiparous women to reduce the number of caesarean sections and associated morbidity.

**Trial registration:**

Clinical Trials NCT04760496, registered on 18th february 2021.

**Project code number:**

APHP200003 / EUDRACT No. : 2020-002640-23. Protocol Version N° 6.0 of 03/09/2024.

## Background

The prevalence of obesity during pregnancy is increasing worldwide. In France, obesity affected 14% of women at the beginning of pregnancy in the last national perinatal survey [1]. The effects of obesity on labour are well known, and include increased duration of active phase of labour and caesarean section rates, that are associated with higher maternal morbidity [2–6].

In order to improve uterine contractions (UC), oxytocin is used during labour, in case of cervical dilation stagnation or failure to progress in the maternal pelvis.

In 2013, in a Swedish prospective observational study of 63.829 nulliparous women with spontaneous onset of labour, Carlhall et al. showed a significant increase in duration of labour in obese patients (9.1 h vs. 8.8 h; *p* < 0.001), an increase in caesarean section rate (10.2% vs. 5.1%, *p* < 0.001) and an increase in the frequency of oxytocin use (50.2% vs. 45.2%, *p* < 0.001) as compared to women with normal weights [5]. Similarly, a French historical study in 31.4851 pregnant women published in 2017 by Deruelle et al. showed an increase in caesarean section rate from 16.4% in patients with a normal body mass index (BMI) to 28.8% in obese patients [7].

In the past few years, induction of labour has become increasingly common. In 2018, Grobman et al. showed in a randomized trial that even for low risk nulliparous women, the frequency of caesarean delivery was significantly lower in the induction group than in the expectant-management group (18.6% vs. 22.2%; relative risk, 0.84; 95% confidence interval (CI), 0.76 to 0.93) [8]. This major randomized trial largely contributed to the fact that practitioners now widely suggest the induction of labour to their patients. In the last national epidemiological survey in France in 2021, 25.8% of women had an induction of labour. Among obese women, this rate is even higher due to more frequent pregnancy-related pathologies, such as gestational diabetes, foetal macrosomia or preeclampsia [8].

In 2020, a literature review highlighted that induced labour and oxytocin use are associated with more caesarean delivery in primiparous women with BMI > 40 kg/m^2^ [9]. In a recent meta-analysis, Ellis et al. showed that caesarean birth was more common among obese women as compared with women of normal weight following labour induction (Mantel-Haenszel fixed-effect odds ratio, 1.82; 95% CI, 1.55–2.12; *p* < 0.001). Maternal obesity was also associated with the use of a higher dose of synthetic oxytocin and a longer time to birth after oxytocin use [10]. In this setting, several factors could explain labour complications. Foetal pelvic disproportion due to foetal macrosomia in obese patients has been traditionally incriminated. The prevalence of macrosomia in the obese women population (9–10%) is known to be higher than in non-obese patients (5–6%) [11]. The uterine over-distension could alter the course of labour by promoting dyskinesia of uterine contractility.

Several biological hypothesis have been made to explain this lower effect of oxytocin in obese women, such as a bigger volume of distribution, a dysfunction of oxytocin receptors due to hypercholesterolemia and a inhibitive effect of leptin production [12].

### Previous studies in the field

In their meta-analysis published in 2013, Kenyon et al. concluded that the use of high-dose oxytocin (greater than 4 mIU/min) vs. low-dose (lower than 4 mIU/min) was associated with a decrease in caesarean section rate without increasing maternal or neonatal morbidity [12]. However, several studies in this meta-analysis were not double-blind, and they did not specifically concern obese women. Unpublished data (F. Pizzagali) of a retrospective study conducted in 219 patients (95 with a normal BMI, 59 over-weighted and 65 obese) showed that the amount of oxytocin required to obtain 5 UC per 10 min increased with BMI from 0.62 IU for normal weight patients to 1.08 IU for obese patients (*p* = 0.02). In the same time, the caesarean section rate was 9.5% for normal weight patient vs. 21.5% for obese patients (*p* = 0.03). There were no differences between the groups concerning the rate of post-partum haemorrhage or neonatal outcomes. In 2011, Garabedian et al., observed in a retrospective study an increase in the use of oxytocin during labour with higher BMIs (BMI ≥ 25 and < 30: OR = 1.38 95% CI [1.28–1.49], BMI ≥ 30 and < 35: OR = 2.05 95% CI [1.79–2.34], BMI ≥ 35: OR = 3.02 95% CI [2.57–3.55]) [3]. Moreover, a recent retrospective study conducted in the United States on 1204 primiparous women reported a linear increase in oxytocin doses used according to BMI without maternal or neonatal deleterious effects [6]. The authors concluded that increasing oxytocin doses in obese patients could be considered to reduce labour duration and the risk of caesarean section.

The hypothesis we made was that higher doses of infused oxytocin could improve uterine contractility, enhance the course of labour and decrease the caesarean section rate. The adverse effects of high-dose oxytocin (uterine hyperactivity and foetal heart anomalies) are scarce and can be avoided by stopping administration of oxytocin, which has a very short half-life. Moreover, there is no evidence that increased oxytocin during labour is associated with an increased postpartum haemorrhage rate.

Hence, it seemed necessary to implement a high-quality randomised trial in obese women with a spontaneous or induced onset of labour, to evaluate whether an increased oxytocin dose (compared to a standard dose) can reduce the rate of caesarean section without increasing maternal or neonatal morbidity. The innovative aspects of this study is the double-blind design, the use of pre-pregnancy weight as an inclusion criterion and the homogeneity of the cohort with only primigravida with a spontaneous or induced onset of labour.

## Methods

### Study design

PROXYMA is a French national multicentre, randomized, double-blind, controlled trial, conducted in 14 maternity units.

### Participants

The inclusion criteria are:

- Age ≥ 18 years
- Primiparous *(no previous childbirth beyond 22 weeks)*
- BMI ≥ 30 kg/m² at the beginning of pregnancy
- Singleton pregnancy
- Spontaneous or induced onset of labour
- Cephalic presentation
- Term ≥ 37 weeks of gestation and < 42 weeks of gestation
- Medical indication and absence of medical contra-indication for oxytocin during labour: inadequate and/or ineffective uterine contraction and/or delayed cervical dilation
- Written consent obtained
- Affiliation to a French social security system

The exclusion criteria are:

- Hypersensitivity to the active substance (oxytocin), to any of its excipients or to latex (risk of cross allergy)
- Medical contra-indication for oxytocin
- Previous coagulation disorders.
- Foetal growth restriction (inferior to 5th percentile)
- Major foetal malformation
- Foetal heart rate abnormalities before use of oxytocin *(at the time of inclusion)*
- History of uterine surgery *(scarred uterus of gynaecological origin)*
- Patient with a disease requiring caesarean section prior to labour *(planned caesarean section before labour)*
- Severe renal failure
- Patients deprived of their liberty (under curatorship or guardianship)
- Participation in another interventional trial of category 1. Patients included in interventional research with minimal risk and constraints may participate in the study after the investigator’s assessment.

### Objectives and endpoints

The main objective of PROXYMA is to compare the effect of higher doses of oxytocin (experimental group) vs. standard doses of oxytocin (control group) on the rate of caesarean sections in obese patients with spontaneous or induced onset of labour.

The secondary endpoints will be:

- to compare this effect on:
  Maternal and labour complications
    ◦ Length of labour
    ◦ Arrest of labour
    ◦ Interruption of oxytocin perfusion and reason
    ◦ Uterine hyper-stimulation
    ◦ Mode of vaginal delivery
    ◦ Reason for caesarean section
    ◦ Post-partum haemorrhage
    ◦ Maternal blood transfusion
    ◦ Volume of oxytocin infusion
    ◦ Oxytocin side effects
  Foetal complications
  Neonatal complications

### Intervention

Oxytocin 5 IU/mL vials will be provided by the Sponsor (AP-HP). The Clinical Trial Department of General Agency of Equipments and Health Products (CTD of AGEPS) will send boxes of oxytocin 5 IU/mL to each pharmacy of study centres.

The oxytocin solution will be prepared by an “out-of-protocol” midwife/nurse and used the same way in both groups: dilution of 1 or 2 vials (depending on the allocated group) in 50 mL (if using an electric syringe) or 500 mL (if using a pump) of 5% glucose serum (G5) bags. The IV infusion will be controlled by pump or electric syringe depending on the habits of the center.

In the control group, 1 vial will be diluted (corresponding to a dose of 5 IU) and will enable the reconstitution of 100 mIU/mL oxytocin solution electric syringes or 10 mIU/mL oxytocin solutions bags.

In the experimental group, 2 vials will be diluted (corresponding to a dose of 10 IU) and will enable the reconstitution of 200 mIU/mL oxytocin solution electric syringes or 20 mIU/mL oxytocin solution bags.

Only the dose of oxytocin will differ between the two groups. For both groups, the infusion rate will start at 1.2 mL/h (dilution in 50 mL electric syringes) or 12 mL/h (dilution in 500 mL G5 bags) and the increment will be made at least every 20 min. The administration will be for an extemporaneous use. The two oxytocin dosing regimens will be the following:

- *Control group (standard dosing)*: 1 vial of 5 IU, with a start infusion rate of 1.2 mL/h or 12 mL/h (corresponding to a dose of 2 mIU/min) depending on the set-up, with equivalent increments (1.2 or 12 mL/h respectively) at least every 20 min, up to a maximum of 12 mL/h (50 mL electric syringes) or 120 mL/h (500 mL bags) respectively, corresponding to a maximum dose of 20 mIU/min.
- *Experimental group*: 2 vials of 5 IU, with a start infusion rate of 1.2 mL/h or 12 mL/h (corresponding to a dose of 4 mIU/min) depending on the set-up, with equivalent increments (1.2 or 12 mL/h respectively) at least every 20 min, up to a maximum of 12 mL/h (50 mL electric syringes) or 120 mL/h (500 mL bags) respectively, corresponding to a maximum dose of 40 mIU/min.

The 4 mIU/min initial dosing in the experimental group was chosen according to the literature. It has been identified as the higher efficient dose with minimum side effects in the general population [12].

Oxytocin dose increments stop when 5 UC per 10 min are obtained, or when cervical dilation is observed. If oxytocin infusion has to be stopped and then resumed (e.g., transient hypertonia or transient foetal heart rate abnormalities), it will be resumed at the initial infusion rates.

The treatment will be given during labour, from prescription until delivery, and will be stopped instantly at birth. The content of several electric syringes/bags may be administered to a single patient within 12 to 24 h before her delivery, depending on the rate of UC and the presence/absence of cervical dilation. The treatment may also be stopped in case of maternal side effects or non-reassuring fetal heart rate.

This randomized double-blind controlled trial design allows highlighting the real effect of using an increased dose of oxytocin (experimental regimen, from 4 to 40 mIU/min) compared to the usual management of labour (standard regimen, from 2 to 20 mIU/min) without any change in the practitioner’s care.

### Implementation of the study and informed consent

In order to match all inclusion criteria, all patients will be included and randomized on the day of labour.

The study will be presented at the time of the labour, during the inclusion visit, in order to validate the inclusion criteria. Information and consent forms will be given to the patient, with a 30 min delay of reflection. If eligible, the patient will then be included and randomized and allocated to either group.

Then, oxytocin solution will be prepared and administered depending on the allocated group, as detailed above (see “*Intervention*” part).

The last visit will be the day of discharge of mother and child. Participation will end on the day mother and child return home. Maternal clinical examination will be performed and data needed to evaluate secondary endpoints will be retrieved. Adverse events will also be collected.

The length of participation will be:

- treatment duration: 1 day
- duration of follow-up period until mother and neonate discharge: max 1 month.

The summary of the chronology of the study is represented in Table 1.

**Table 1:** Summary of the chronology of the study

Actions	Inclusion visit: day of labour (1 day)	End of study (mother and neonate discharge)
Verification of inclusion and exclusion criteria	X	
Information	X	
Signature of consent	X	
History	X	
Clinical examination	X	X
Randomization	X	
Administration of treatment (oxytocin)	X	
Mother data collection	X	X
Newborn data collection	X	X
Adverse events	X	X

### Collection of data

Data will be collected in an electronic case report form (e-CRF) by each main investigator of the different centres. Data collection for this study covers the period from October 2021 to February 2026.

The computer file used for this research is implemented in accordance with French (amended “Informatique et Libertés” law governing data protection) and European (General Data Protection Regulation – GDPR) regulations.

This research is governed by the CNIL (French Data Protection Agency) “Reference Methodology for processing personal data used within the scope of health research” (amended MR-001). AP-HP, as sponsor of the research, has signed a declaration of compliance with this “Reference Methodology”.

### Sample size

Based on Audipog data (Réseau sentinelle AUDIPOG, 2019) the caesarean section rate in obese primiparous women at term requiring oxytocin perfusion is around 20%. Our objective is to be able to show a 7% (one third) difference in caesarean section rates among these women, according to the oxytocin dose infused, with a power of 80% and a type I risk of 5%. Thus, 441 women must be included in each group (experimental and control).

To determine the number of women to be screened for consent and the number of centres to be involved, we used the following figure coming from French or European studies [13]: 98% of pregnancies are singleton.

- among them, 45% are primiparous
- among them, 10% of women are obese
- among them, 56% have a spontaneous onset of labour
- among them, 70% require oxytocin perfusion.

Finally, we have considered that the percentage of women that will disagree to participate in the trial could be as high as 40% and that 10% of women who initially agreed will eventually refuse or be lost to follow-up. This results in a total of 94,500 deliveries in participating centres, corresponding to 4,167 primiparous obese women and then to 882 women receiving oxytocin perfusion who agree to be randomly assigned to one of the compared groups. Nationally, a 7% reduction in caesarean sections in obese primiparous women requiring oxytocin means about 1,250 fewer caesarean sections per year in this population.

### Identification of participants, randomisation and blinding methods

The participants in this research are identified as follows: site number (3 digits) - sequential enrolment number for the site (4 digits) - surname initial - first name initial. This reference number is unique and will be used for the entire duration of the study.

The randomization between the two oxytocin dosing regimens takes place at the time of the oxytocin prescription, meaning during labour. Patients are randomized to one of the two groups in a ratio of 1:1, by blocks of random size and with stratification on the centre (maternity hospital). Inclusion are centralized and performed electronically with an Interactive Web Response System (IWRS): CleanWeb^®^ (Telemedecine). The investigator will connect to the IWRS and confirm that the patient meets all inclusion criteria and has no exclusion criteria. The treatments are allocated and randomized by randomization envelopes. The randomization group is not communicated to the investigator or the patient, to preserve the double-blind design. The treatment number specified on the outside of the sealed randomization envelopes is filled in the CleanWeb^®^ e-CRF later by the clinical trial technician of each investigator centre.

The patient is assigned to a numbered envelope, containing her randomization group. This envelope must not be opened by the investigator, but only by an “out-of-protocol” nurse/midwife, who is in charge of the oxytocin dosage preparation but is not investigator of the study nor involved in patients care. All chosen centres have a large team on call 24/24 that allows one midwife/nurse to be available as “out-of-protocol”. The randomization group must not be communicated by the “out-of-protocole” nurse/midwife to the investigator, any medical personnel or patient involved in the study in order to preserve the double-blind design. The oxytocin electric syringes or bags is prepared outside of the delivery room, and the investigator (or any medical personnel involved in the study) and the patient will not know the oxytocin dosage. Oxytocin preparation is part of standard obstetrical care, yet all “out-of-protocole” midwifes/nurses are trained to protect blinding.

Unblinding can be requested for any reason considered essential by the investigating physician.

### Statistical methods

Data management and statistical analysis will be performed by the Unité de Recherche Clinique Paris Saclay.

- Descriptive analysis

The characteristics of the women at the time of randomization will be described separately for the two randomized groups by providing mean and interquartile range (IQR) for quantitative variables and number and percentages for qualitative variables. No comparative statistical test will be provided (a priori, the null hypothesis is always true thanks to the randomization). However, variables with a substantial difference between groups will be identified for further adjustment.

b) Analysis of the primary outcome

The primary outcome will be analysed by comparing the percentages of caesarean sections in the two groups. To take into account the multicentre nature of the trial, analyses will be adjusted for centres using logistic regression. The analysis will be conducted on an “intention to treat” (ITT) basis in which women remain in their initial group, as usual in superiority trials. Complementary analyses will be done by adjusting for BMI and for variables with a substantial difference between groups. Additional sensitivity analyses will be done on a “per protocol” (PP) basis. There will be no interim analyses.

c) Analysis of secondary outcomes.

Secondary outcomes results will be considered as exploratory and as a way to comment or explain the results of the primary outcome. The comparisons between groups will be adjusted for centres and for variables with a substantial difference between groups. No corrections for multiple tests will be made.

Missing data will not be replaced. Analysis will be done in a complete-cases basis.

The statistical analysis will follow a Statistical Analysis Plan (SAP) which will have to be validated by the coordinating investigator, the methodologist and the biostatistician before the database lock (at the latest). Any modification posterior to the database lock will lead to a post-hoc SAP.

## Discussion

### Potential implication of the findings

A major issue in obstetrics is how to reduce the number of caesarean sections especially in a high risk group of patients. In the general population, a caesarean section during the first delivery alters the obstetrical prognosis for future pregnancies by increasing the risk of repeat caesarean section, poor placental insertion (placenta accreta) or uterine rupture. For obese women in particular, caesarean sections expose them to significant morbidity. Moreover, loco-regional anesthesia presents technical difficulties in these women: increased risk of failure of needle insertion, increased rate of breach of the dura mater and intravascular catheterization. If general anaesthesia is required, obesity is a major risk for difficult intubation, gastric inhalation and ventilation difficulty [14]. Moreover, sometimes long and difficult caesarean sections in such patients may impact neonatal outcomes in case of emergency, and may lead to more frequent maternal events such as anemia, infections and thrombo-embolic events [15–17].

Hence, the present trial will compare two strategies with different oxytocin doses in obese women in order to reduce the number of caesarean sections and its associated morbidity. Its results will provide clinicians with better knowledge on the management of labour in obese women.

## Data Availability

No datasets were generated or analysed during the current study.

## Abbreviations

ANSM: Agence Nationale de Sécurité du Médicament
AP-HP: Assistance Publique-Hôpitaux de Paris
BMI: Body mass index
CI: Confidence interval
CNIL: Commission Nationale Informatique et Libertés
CPP: Comité de Protection des Personnes
CTD of AGEPS: Clinical Trial Department of General Agency of Equipments and Health Products
e-CRF: electronic case report form
G5: Glucose 5%
IQR: Interquartile range
ITT: Intention to treat
IWRS: Interactive Web Response System
SAP: Statistical analysis plan
UC: Uterine contractions
